# Spore development and nuclear inheritance in arbuscular mycorrhizal fungi

**DOI:** 10.1186/1471-2148-11-51

**Published:** 2011-02-24

**Authors:** Julie Marleau, Yolande Dalpé, Marc St-Arnaud, Mohamed Hijri

**Affiliations:** 1 Université de Montréal, Département de sciences biologiques, Institut de recherche en biologie végétale, 4101 rue Sherbrooke Est, QC, H1X 2B2, Canada; 2 Agriculture and Agri-Food Canada, 960 Carling Ave. Ottawa, On, K1A 0C6, Canada

## Abstract

**Background:**

A conventional tenet of classical genetics is that progeny inherit half their genome from each parent in sexual reproduction instead of the complete genome transferred to each daughter during asexual reproduction. The transmission of hereditary characteristics from parents to their offspring is therefore predictable, although several exceptions are known. Heredity in microorganisms, however, can be very complex, and even unknown as is the case for coenocytic organisms such as Arbuscular Mycorrhizal Fungi (AMF). This group of fungi are plant-root symbionts, ubiquitous in most ecosystems, which reproduce asexually via multinucleate spores for which sexuality has not yet been observed.

**Results:**

We examined the number of nuclei per spore of four AMF taxa using high Z-resolution live confocal microscopy and found that the number of nuclei was correlated with spore diameter. We show that AMF have the ability, through the establishment of new symbioses, to pass hundreds of nuclei to subsequent generations of multinucleated spores. More importantly, we observed surprising heterogeneity in the number of nuclei among sister spores and show that massive nuclear migration and mitosis are the mechanisms by which AMF spores are formed. We followed spore development of *Glomus irregulare *from hyphal swelling to spore maturity and found that the spores reached mature size within 30 to 60 days, and that the number of nuclei per spores increased over time.

**Conclusions:**

We conclude that the spores used for dispersal of AMF contain nuclei with two origins, those that migrate into the spore and those that arise by mitosis in the spore. Therefore, these spores do not represent a stage in the life cycle with a single nucleus, raising the possibility that AMF, unlike all other known eukaryotic organisms, lack the genetic bottleneck of a single-nucleus stage.

## Background

The Arbuscular Mycorrhizal Fungi (AMF) are a group of root-inhabiting, symbiotic organisms that are widely distributed geographically and are among the most common soil fungi. AMF form symbioses with the roots of approximately 80% of all vascular plant species [1]. These fungi offer a wide variety of host benefits, the most well-known being an increase of mineral uptake, particularly of phosphorus [2], a better drought tolerance through increased water uptake [3,4] and a higher resistance to root pathogens [2]. Mycorrhizal plants also experience improved nodule function in the case of legumes [5] and better soil structure, due to the ability of the fungi to bind soil particles and decrease soil erosion [6].

AMF have existed unchanged morphologically for at least 460 million years, despite lacking sexual reproduction [7]. AMF are coenocytic organisms that have evolved to be multigenomic, possessing a large amount of genetic variation for ribosomal DNA [8-12], non-coding regions [13] and also for protein-coding genes [12,14,15] not only between individuals, but among nuclei within an individual (one 'individual' refers here to a single spore). Reproduction occurs by asexual spores that contain hundreds or even thousands of nuclei [16,17], and these spores are the only form under which species can be identified morphologically, although AMF can be also identified using molecular markers. The ability of spores to germinate is a prerequisite for the establishment of mycorrhizal symbiosis for many AMF taxa. However, while the developmental process leading to the accumulation of nuclei within spores has been recently reported to occur by the transport of numerous nuclei into the developing spore in the AMF *Glomus etunicatum *[18], whether or not mitosis happens within spores is not known. Fungal mitosis has been accurately described and illustrated mainly in ascomycetes and basidiomycetes and is usually intranuclear (reviewed in Aist and Morris, 1999 [19]) because the nuclear envelope remains intact, often until anaphase B. Coenocytic fungi such as zygomycetes have also an intranuclear mitosis. However, the coexistence of numerous nuclei within a common cytoplasm and mitosis phases that are not easily distinguishable causes many difficulties in studying mitosis in coenocytic fungi, although exceptions have been reported in *Basidiobolus ranarum *[20]. To test whether mitosis is involved during sporulation in AMF, we used mitosis inhibitors. Many mitosis inhibitors have been successfully tested on AMF. For example, Aphidicolin, which is a specific DNA polymerase α inhibitor that blocks the cell cycle at early S phase, was used to study nuclear division in AMF during *in vitro *development [21]. Carbendazim, which prevents microtubule formation and inhibits mitosis in fungal cells, was also used to investigate phosphorus transport and succinate dehydrogenase activity in three AMF [22]. We chose the use of aphidicolin in our experiment because it has been shown to inhibit efficiently mitosis in AMF without any effect on spore germination [21].

To investigate the mechanisms of AMF spore formation in other taxa, and by extension, the amount of genetic material inherited by AMF offspring, we first counted the number of nuclei in live spores belonging to four different AMF taxa. All these taxa were grown in *in vitro *culture with *Agrobacterium rhizogenes *T-DNA transformed-carrot roots and were examined using confocal microscopy with a high z-resolution for three-dimensional (3D) reconstructions, which allows a direct counting of nuclei and visualization of their 3D architecture. We addressed five specific questions: 1) what is the correlation between spore diameter and the number of nuclei per spore? 2) Is there any heterogeneity in the number of nuclei among spores of similar diameter? 3) What is the mechanism of spore formation in the AMF *G. irregulare*? 4) What is the minimum number of nuclei required to make up a viable spore that can germinate and establish a functional symbiosis in *G. irregulare*? 5) Do the extraradical phases of AMF lack a single nuclear stage? These questions are of fundamental importance to our comprehension of AMF reproduction by sporulation, and their answers will bring a new level of knowledge to AMF genetics and evolution. Insight into AMF genetics is the cornerstone on which understanding the role of mycorrhizal symbiosis in nature will be based.

## Results

### Live cell imaging of AMF spores

**Figure 1:**
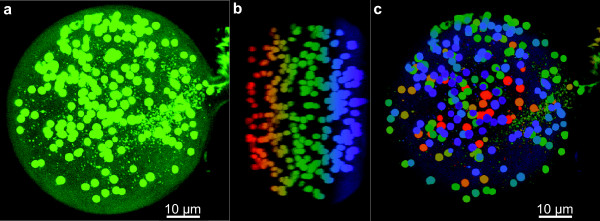
**Live cellular imaging of AMF**. Spore of *G. diaphanum *visualized by confocal microscope showing 208 nuclei stained with SytoGreen fluorescent dye. **A**, 2D (x, y) merged image with nuclei shown as green spots. **B**, 2D (y, z) merged digital image colour-indexed on z-depth from red to violet. **C**, 2D (x, y) merged digital image colour-indexed: red colour on the bottom and violet on the top to facilitate nuclear counting. Panels **A **and **C **are a maximum intensity projection of 260 optical sections with z-resolution of 0.15 μm. Scale bar represents 10 μm.

**Figure 2:**
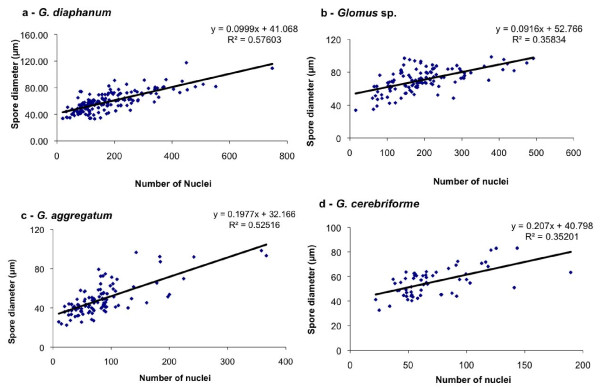
**Number of nuclei per spore**. The number of nuclei in juvenile and mature spores plotted against spore diameter of *G. diaphanum *(y = -162.53 (*P *= 0.00) + 5.76x (*P *= 0.00), *R^2 ^*= 0.57). The diameter of spores plotted against the number of nuclei per spore. **A**, *G. irregulare *(n = 113); **B**, *G. aggregatum *(n = 105) and **C**, *G. cerebriforme *(n = 60). *G. cerebriforme *spores had the smallest number of nuclei per spore and were also the smallest in size, while *G. diaphanum *spores had the largest number of nuclei per spore. There is a positive linear relation between the number of nuclei per spore and spore diameter: *G. irregulare *(y = -81.09 + 3.91x, R^2 ^= 0.3583), *G. aggregatum *(y = -45.69 + 2.65x, R^2 ^= 0.5252) and *G. cerebriforme *(y = -21.35 + 1.66x, R^2 ^= 0.3425). All slopes were statistically significant at *p *< 0.05.

We used a novel high-accuracy method for determining the number of nuclei in live spores (Figure 1, Additional Files 1 and 2) and found that in 166 live spores of *G. diaphanum*, the number of nuclei per spore ranged from 20 to 748 for diameters of 33 to 109 μm, respectively. Figure 2 shows the diameter of spores plotted against the number of nuclei per spore of *G. diaphanum *(n = 166); *G. irregulare *(n = 113); *G. aggregatum *(n = 105) and *G. cerebriforme *(n = 60). *G. cerebriforme *spores had the fewest nuclei per spore and were also the smallest in size, while *G. diaphanum *spores had the greatest number of nuclei per spore. We found a positive linear relation between the number of nuclei in a given spore and its diameter for all AMF taxa: *G. irregulare *(y = -81.09 + 3.91x, R^2 ^= 0.3583), *G. aggregatum *(y = -45.69 + 2.65x, R^2 ^= 0.5252) and *G. cerebriforme *(y = -21.35 + 1.66x, R^2 ^= 0.3425). All slopes were statistically significant at *p *= 0.05.

### Heterogeneity of the number of nuclei per spores

**Figure 3:**
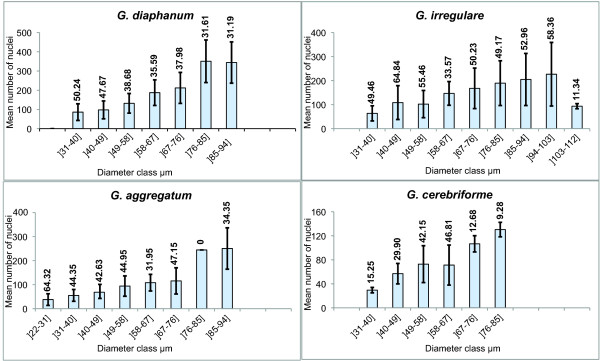
**Heterogeneity of the number of nuclei per spore**. The number of nuclei per spore compared with spore diameter grouped by classes for four AMF taxa. Coefficients of variation are shown in bold.

We also compared the number of nuclei per spore and the spore diameter of different classes for each of our four AMF taxa. We found a high heterogeneity in the number of nuclei per spore of the same diameter classes (Figure 3 and Additional File 3). For example, the number of nuclei in *G. irregulare *spores varied by a factor of nearly 5 in the diameter class 94-103 μm.

### Mechanism of spore formation in *G. irregulare *and *G. diaphanum*

We used two independent, but complementary, imaging experiments to examine the nuclear inheritance process. In a two-compartmented *in vitro *system [23], *G. irregulare *was grown on transformed carrot roots that were restricted to one compartment (proximal). Only the fungus was permitted to grow on to the second compartment (distal) containing the same media lacking the sugar and complemented with aphidicolin [17]. The formation of spores over time was monitored in the distal compartment. We found that while aphidicolin did not inhibit spore formation or hyphal growth, the number of newly produced spores was reduced by 14-fold in comparison to the control (Additional File 4). We then examined the number of nuclei per spore and the diameter of spores that were produced in the distal compartment for both aphidicolin and control treatments. The aphidicolin had no significant effect on the spore diameter (W = 782, *p *= 0.753), but reduced the number of nuclei per spore by 54.86% (W = 1207.5, *p *= 0.00001) (Additional File 5). On average, nuclear number per spore was 55.23 ± 40.31 SE, (n = 22) and 168.30 ± 90.66 SE, (n = 165) for aphidicolin and control treatments, respectively.

**Figure 4:**
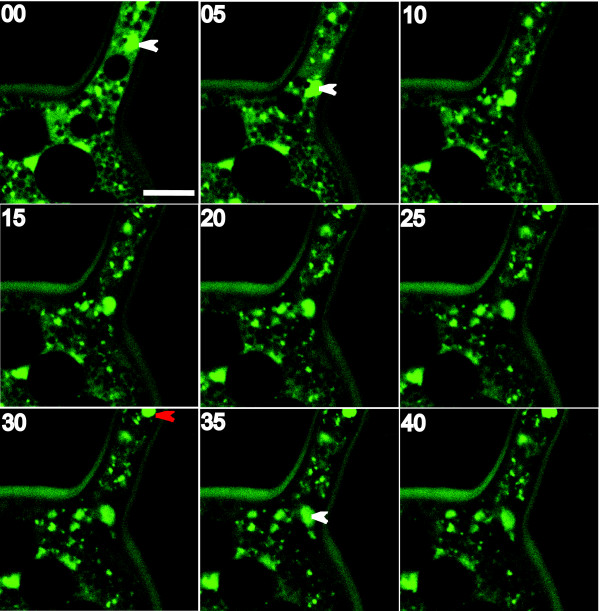
**Time-lapse series on AMF live spores**. Panels represent 5 min intervals in a time-lapse sequence monitoring nuclear migration into a developing spore of *G. diaphanum *by confocal microscopy. SytoGreen stained nuclei move unidirectionally into the spore. Images were acquired on xyt mode. Arrowheads indicate the first (white) and second (red) nucleus entering the spore. Note that some nuclei are out of focus in the xy optical section and can be difficult to visualize. Scale bar on the top-left panel represents 10 μm. Numbers on the top left of each panel are minutes.

To confirm this nuclear migration we next made direct observations of individual nuclei in live cells in real time. Time-lapse series taken with a confocal microscope of *G. diaphanum *spores clearly shows nuclear migration from the hypha into the developing spores (Figure 4 and Additional File 6).

### Spore formation over time and spore viability

**Figure 5:**
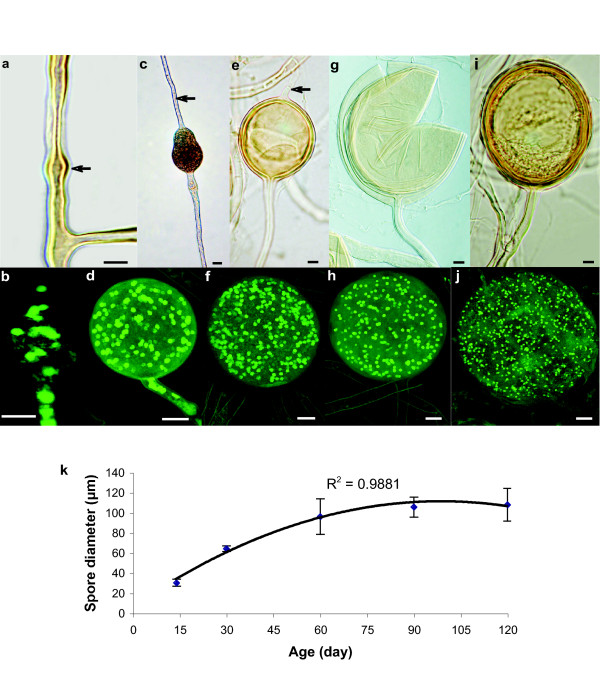
**Spore development over time**. **A **and **B**, Intercalary swellings along the double-walled hypha give rise to a spore primordium (arrow in **A**). **C **and **D**, 15 day-old juvenile spore with subtending hypha and a septum in the mother hypha (arrow in **C**). **E **and **F**, Intercalary 30 day-old spores with subtending hypha and bi-layered spore wall. **G**, Crushed 60 day-old spore with subtending hypha and separating spore wall layers. The apical portion of the mother hypha has emptied, septated and detached from the spore (arrow in **E**). **H**, 60 day-old spore observed with a confocal microscope. **I **and **J**, 90 day-old mature spore still attached to subtending hyphae. The images in panels **A**, **C**, **E**, **G **and **I **were taken using a DIC optical microscope. The merged images in panels **B**, **D**, **F**, **H **and **J **were taken using a confocal microscope where nuclei were stained with SytoGreen fluorescent dye. Scale bars represent 10 μm. **K**, Spore diameter plotted against spore age using a quadratic model (y = -0,0107X^2 ^+ 2,1243X + 5,9132).

We followed spore formation of the AMF *G. irregulare *over time for 120 days using optical microscopy (Figure 5). We began with spore primordia (time 0) (Figure 5A-B) and followed their development process at different intervals (0, 15, 30, 60 and 90 days; Figure 5A-J). We found that fast spore growth occurs during the first 30 days after hyphal swelling, with a 10-fold increase in spore volume between 15 and 30 days of growth (Table 1) and that spore size increases slow down during the subsequent 60 days. In addition, an increase of the number of nuclei per spore over time was observed when the number of nuclei was assessed by confocal microscopy (Figure 5B, D, F, H and 5J). More importantly, while approximately 90% of the spores, from 15 days old and older, were viable as assessed by vital dye staining, only spores that were at least 30 days old were able to germinate and to regenerate an *in vitro *functioning colony when associated with transformed carrot roots (Table 1). The number of nuclei in these 30 day-old spores ranged from 65 to 222 with a mean of 153.33 ± 46.16 SE (n = 18). The number of nuclei in 15 day-old spores, that were viable but could not germinate, ranged from 16 to 101 with a mean of 65.09 ± 32.1 SE (n = 11). We examined the number of nuclei in mature spores that failed to germinate and observed that many of them contain no nuclei or degenerating nuclei (Additional File 7). However, some non-germinating spores had the same number of nuclei as others that were able to germinate.

**Table 1:** A, Spore volume increase of *in vitro *differentiated spores of *G. irregulare *over time (n = 25); B, Percent spore viability and percent spore germination of *in vitro *differentiated spores of *G. irregulare *over time (n = 25, for each selected age category)

Age of spores	Mean spore diameter/radius (μm)	Spore volume (mm^3^)	Viability (%)	Germination (%)
14 days	30.4/15.2	0.000015	90	0
30 days	64.2/32.1	0.000139	90	40
60 days	96/48	0.00046	90	38
90 days	105.7/52.8	0.00061	95	50

### Nuclear stage of extraradical phase

A careful examination of spores, runner hyphae and fine branchings from five independent extraradical symbiotic mycelia of *G. diaphanum *has shown that the number of nuclei per mycelium biomass changed with the morphological structure. There were more nuclei per spore than were observed in 100 μm of either runner hyphae or fine branchings, a length of the same magnitude as the spore volume. However, considering their small diameter, the number of nuclei per unit volume of mycelium was significantly higher in the runner hyphae and fine branchings compared with spores (Table 2). The formation of septa was not observed in runner hyphae but was noted in 32% of the fine branchings, with numbers of nuclei varying from 0 to 18.5 per 100 μm of hyphal length. Interestingly, compartments containing only a single nucleus were never observed.

**Table 2:** Number of nuclei in the different architectural structures forming the extraradical mycelium of *G. diaphanum *(n = 21 spores, 20 RH and 22 FB)

Architectural structure^1^	Diameter × length^2 ^(μm)	Nb of nuclei	Concentration ^3 ^(×μm^3^)
Spore	72.6	282/spore	0.00147419 a
RH	6.9 × 157.3	10.3/100 μm	0.00306436 b
FB	3.3 × 131.0	5.8/100 μm	0.00851064 b

^1 ^Structures are spores, runner hyphae (RH) and fine branching (FB).

^2 ^Diameter (all) and length (RH, FB) or the structure portion used to count the nuclei.

^3 ^Concentrations of nuclei followed with a different letter are significantly different between structures by a **Tukey-Kramer **HSD test.

## Discussion

The number of nuclei in spores has been previously estimated by several methods with widely divergent results [16]. For example, using a model in which nuclei occur as a single layer around the periphery of the cytoplasm [24], the number of nuclei in *Gigaspora margarita *spores was estimated at about 20,000, while a different method based on digital image measurements estimated the number of nuclei per spore to be about 2,000 [17]. Here we used confocal microscopy with a high z-resolution for three-dimensional (3D) reconstructions, which allow a direct counting of nuclei and visualization of their 3D architecture in live spores. As far as we know, this is a novel rapid and high-accuracy method for determining the number of nuclei per spores in AMF. Recently, Jany and Pawlowska [18] have reconstructed 3D images of the AMF *G. etunicatum *using deconvolution, a computational based-method used to reduce out-of-focus fluorescence in 3D microscope images.

We found a surprisingly high heterogeneity in the number of nuclei per spore of different diameter classes. The number of nuclei in *G. irregulare *spores varied by about 5-fold in the diameter class 94-103 μm. Clearly, the spores of the AMF used here do not have a constant number of nuclei.

We used two independent but complementary imaging experiments based on aphidicolin treatment and time-lapse live cellular imaging to examine the nuclear inheritance process in *G. irregulare *and *G. diaphanum*. We used *G. irregulare *in aphidicolin treatment because it is fast growing under *in vitro *culture. However, *G. diaphanum *was chosen for time-lapse experiments because its spores contained the higher number of nuclei. When tested on AMF spores, aphidicolin does not inhibit spore germination of the AMF *G. margarita*, but rapidly reduces the rate of hyphal growth and arrests growth after four days, owing to efficient inhibition of DNA synthesis [17]. We tested aphidicolin on yeast cells and found that no mitosis occurred in samples grown in media complemented with aphidicolin whereas optical density (DO) increased over time in samples without aphidicolin (data not shown). When spore formation was monitored over time in the distal compartment, we found that the aphidicolin did not inhibit the spore formation or the hyphal growth, but the number of newly produced spores was dramatically reduced. Aphidicolin had no significant effect on the spore diameter (*F*_1.88 _= 0.02, P = 0.90), but reduced the number of nuclei per spore by approximately three-fold (*F*_1.88 _= 15.18, P ≤ 0.0002). Since the nuclei observed in the spores differentiated in the distal compartment (containing aphidicolin) could not have been produced by mitosis in the newly formed spores, they must have migrated from the proximal compartment, through hyphae, into the developing spore. Considering that the average nuclear number per spore treated with aphidicolin was approximately three times less than that of the control, at least one mitotic cycle must occur that during spore development, allowing a duplication of the migrated nuclei. To confirm this nuclear migration, we made direct observations of individual nuclei in live cells in real time. Time-lapse series taken with a confocal microscope of the AMF *G. diaphanum *spores clearly show nuclear migration from the hypha into the developing spores. In total, we observed 534 live AMF spores of four taxa, all of which showed continuous connections between hyphal and spore cytoplasm without any apparent septa (Additional File 8). These results support the hypothesis that AMF spores are formed by a massive nuclear migration and are in agreement with results reported using *G. etunicatum *[18]. Interestingly, these nuclei could potentially carry different genomes, as there is considerable evidence suggesting that AMF contain populations of genetically different nucleotypes coexisting in a common cytoplasm [12,14,15,25-28]. In addition, genetic exchange has only recently been demonstrated to occur among AMF isolates that are genetically distinct [29]. There is as well recent evidence showing that genetic exchange and segregation in AMF significantly affects the plant response [27].

Taken together, our results clearly show that AMF spores accumulate up to thousands of nuclei by nuclear migration and mitosis. However, one potential consequence of this mechanism is the loss of the genetic diversity by drift-driven processes. During sporulation, the evolutionary process of change in the nucleotype frequencies (or genome frequencies) of a nuclear population from one generation to the next is due to the phenomenon of probability, in which pure chance events determine which nuclei within the overall nuclear population will be carried forward while others disappear. Two strategies could be envisaged to counter a potential loss of genetic diversity in AMF, either a selective sampling of certain nucleotypes from the overall population, or random selection coupled with a mechanism to ensure that spores contain adequate numbers of nuclei. Although previous models have demonstrated that hyphal fusion and nuclear exchange is an important mechanism for the maintenance of high nuclear diversity [30], this has been also reported to occur between genetically different isolate of the AMF *G. intraradices *[29]. Recombination has been reported to occur in AMF [31] but at low frequency and thus is likely to contribute significantly in maintaining the unusual high genetic diversity observed frequently within AMF isolates [12,28]. It has been reported that recombination repair of double-strand DNA breaks in mitotic cells is associated with a 100-fold higher mutation rate in *Saccharomyces cerevisiae *[32]. In addition, *S. paradoxus *has a low frequency of recombination rate but maintains a completely outbreeding population structure [33].

We also wished to estimate the minimum number of nuclei to make up a viable spore (i.e., able to germinate). To address this issue, we followed spore formation of the AMF *G. irregulare *over time for 120 days using optical microscopy. We examined the number of nuclei in mature spores that failed to germinate and observed that some had the same number of nuclei as others that were able to germinate. However, while this runs counter to the idea that spore germination requires a minimum number of nuclei, it is possible that this minimum number may vary due to other factors such as spore maturity or dormancy. Interestingly, *G. etunicatum *spores reached their mature size within 2-3 days [34], while *G. irregulare *isolate DAOM-197198 spores took 30 to 60 days to reach their mature size (Figure 5 and Additional Files 9 and 10). This is surprising because *G. irregulare *is a very fast growing AMF and therefore used as commercial inoculant sold worldwide while the *G. etunicatum *isolate NPI used in our laboratory has an extremely slow growth rate.

We examined various stages of the extraradical phases of *G. irregulare *including the symbiotic phase (running hyphae, branching hyphae and developing spores) and the resting phase (mature spores) and did not observe any structure with a single nucleus (Table 2). Although our observations were based on *in vitro *laboratory cultures, a single cell with a single nucleus has also never been found in nature, even though AMF are well studied in natural ecosystems. Our results provide no support for sexual reproduction or a single-nucleus stage in the life cycle of AMF, which suggests that asexual reproduction dominates in these fungi. However, demonstrations of hyphal fusion between individuals [29] and reports of recombination [13,31,35] indicate that genetic exchange and recombination also contribute to the population structure of these fungi. It has been hypothesized that auxiliary cells in *Gigaspora *species might represent vestiges of relict reproductive structures [36] but without evidence of karyogamy. Tommerup [37,38] has reported the formation of what was interpreted as zygospores in *Gigaspora decipiens *in 0.24% of hyphal contacts in a monosporal-grown mycelium, while all other contacts resulted in vegetative growth with or without anastomosis formation. Except for this case, to our knowledge gametangial formation has never been reported elsewhere despite many descriptions of the extramatrical mycelia architecture [39-42] and an extensive literature on intraradical colonization [43,44].

## Conclusions

We reported that AMF spores contain a high heterogeneity in the number of nuclei among sister spores and showed that massive nuclear migration and mitosis are the mechanisms contributing to the sporulation in AMF. The high levels of genetic variation within individuals combined with the large number of nuclei in AMF spores may thus be the evolutionary strategy adapted by AMF in order to reconcile their multigenomic organization with the need to remain adaptable to diverse micro-environmental changes. The finding that mitosis occurs in spore formation in AMF brings a new level of understanding of reproduction and nuclear inheritance processes in this major eukaryotic symbiosis.

## Methods

### Fungal material

The four AMF species, *Glomus irregulare *(isolate DAOM-197198, previously known as *G. intraradices *[45]), *G. cerebriforme *(DAOM-227022), *G. aggregatum *(strain 2101-sp) and *G. diaphanum *(DAOM-229456) used in our studies, were obtained from the Glomeromycota *In vitro *Collection (GINCO). These strains were co-cultured with Ri T-DNA-transformed carrot roots (*Daucus carota *L.). AMF strains and transformed carrot roots were cultured and maintained on a minimal (M) medium [46] solidified with 0.4 w/v Gellan gum at 25°C.

### Confocal microscopy

Arbuscular mycorrhizal fungal spores were freshly collected from plates by dissolving the Gellan gum in citrate solution [47], washed with sterile water and immediately stained with 2% (v/v) of SytoGreen 13 live fluorescent dye (Invitrogen, Canada), for 30 min at room temperature and in darkness. Stained spores were mounted in an 80% glycerol solution and visualized using a Zeiss LSM 5 DUO confocal microscope equipped with Piezo (xy) stage. Specimens were imaged with an objective LCI Plan-Neofluotar 63× (NA 1.3) in water and glycerine immersion and using an argon laser at 488 nm. For each specimen, z-stacks (approximately 150 optical sections depending on the spore size, with an interval of 0.2 μm) were recorded. Z-stacks were then merged into 2D images in which optical sections were colour indexed in order to facilitate manual counting of nuclei. Time-lapse experiments were performed using (x, y, t) mode and 10 optical sections were collected at each 5 min interval for each series.

### Aphidicolin experiments

Two compartment 100 × 15 mm Petri dishes [23] were used in this experiment with the AMF *G. irregulare*. One compartment was filled with 25 ml of M medium to the top level of the dividing wall, while the other compartment was filled with only 10 ml of M medium lacking sugar and complemented axenically with 150 μM of aphidicolin [17] in DMSO (Sigma, Canada) after it had been autoclaved and allowed to cool to 45°C. After solidification of the medium, an additional 1 ml of sugarless M medium containing aphidicolin was deposited on the dividing wall in the sugarless compartment, placed at an angle in order to form a bridge and facilitate hyphal crossing between the two compartments but without continuous connection between growth media [23]. Control plates were prepared in the same conditions, except the second compartment was filled with sugarless M medium complemented with an equal volume of DMSO only. *G. irregulare *colonized transformed carrot roots were added to the compartment containing sugar. Plates were then incubated in the dark at 25°C until the mycelium had crossed the dividing wall and grown on the second compartment. The cultures were examined weekly. The number of newly formed spores was counted weekly on the second compartment containing aphidicolin or DMSO as a control. After 3 months of culture, spores from this compartment were collected by dissolving the gel, rinsed with sterile and stained with SytoGreen as described above. Spores were examined as described in the confocal microscope section.

### Spore selection and measurement over time

Hyphal swellings differentiated along AMF mycelium filaments on the fungal side of two-compartment plates were marked by pen on the plate bottom and their development followed under episcopic light with a dissecting microscope (Nikon SMZ 10A). Twenty-five marked 15, 30, 60 and 90 day-old spores, respectively, were recovered manually from the fungal colonies and mounted on microscopic slides in PVLG mounting medium [48]. Cross diameters of mounted spores were measured at magnification 600X (Nikon Eclipse 800), photographed (Digital camera Nikon Coolpix 950) and spore wall development sequentially described.

### Viability of spores

Spore viability was estimated by the MTT 3-(4,5-dimethylthiazol-yl-2,5-diphenyl-2H-tetrazolium bromide) vital stain procedure [49]. Using separate cavity slides, 25 spores were treated with MTT for 40 hours to allow maximum staining response. Treated spores were observed under dissecting microscope. Red and blue stained spores were considered viable. At 15 days old, the majority of spore primordia tested positive for viability and the level of viability remained unchanged throughout the spore maturation period (15-90 days old). The high level of viability and the uniformity of results may be attributed to the fact that spores were produced *in vitro*, under uniform environmental conditions and with no growth constraints. As such, the fungal material may have been less susceptible to mortality or abortion compared to spores isolated directly from soil [50].

### Germination potential of spores

Twenty-five spores of each selected age category (15, 30, 60, 90 days) were extracted from two-compartment *in vitro *cultures, deposited on H_2_O agar medium (pH 6.0) and incubated for a maximum of 30 days at 27°C in darkness. Spores bearing germinating hyphae longer than 150 μm were considered germinated. Thirty day-old spores already showed a 40% germination potential and the capacity to regenerate an *in vitro *functioning colony when associated with transformed carrot roots. This germination potential, whatever the level of spore maturity, never reached more than 50% of the spores, even in those considered viable according to the MTT staining test. The 30 days incubation period chosen may not have been sufficient to demonstrate the full germination capability. However, most of the spore germination observed occurred in the first 3-6 days after incubation, indicating a sufficient maturity to undergo the germination process.

### AMF extraradical mycelium

The extraradical symbiotic mycelium of AMF exhibits three distinguishable morphological structures: large-diameter, relatively unbranched, thick-walled hyphae called runner hyphae (RH) [40], small-diameter thin-walled branched hyphae called arbuscule-like structures [51] or fine branching (FB) [52] and spores. To assess for the presence of uninucleate structures, the mycelium of *G. diaphanum *was carefully examined. Mycelia samples were harvested from five different plate cultures by dissolving the gellan gum in citrate buffer [47], washed with sterile water and immediately stained with 10 μM SytoGreen 13 for 60 min at 35°C in the dark. Stained mycelia were mounted in 50% glycerol solution on a glass plate and 20-22 RH, FB and spores were randomly chosen and visualized using a Zeiss confocal microscope as described above. The nuclei were counted and the volume was calculated from the diameter (for all structures) and length (for RH and FB).

### Statistical analyses

Statistical analyses were conducted using the using JMP 7.0 and Statgraphics Plus v3 statistical software. Since no transformation produced a normal distribution of the residuals, Kruskal-Wallis non-parametric one-way analysis of variance by ranks were conducted to determine the effect of aphidicolin on spore diameter and number of nuclei per spore, and to compare the number of nuclei between morphological structures of *G. diaphanum *symbiotic extraradical mycelia, while *a posteriori *comparisons between means were done using Tukey-Kramer honestly significant difference tests. In order to determine the relationship between the number of nuclei per spore and the diameter of spores, a linear regression model (y = ax + b) was fitted to the number of nuclei (y) with respect to spore diameter (x) for each AMF species.

## List of abbreviations

AMF: Arbuscular Mycorrhizal Fungi; 3D: three-dimensional; DAPI: 4',6-diamidino-2-phenylindole; MTT: 3-(4,5-dimethylthiazol-yl-2,5-diphenyl-2H-tetrazolium bromide); RH: runner hyphae; FB: fine branching; DMSO: Dimethyl sulfoxide; DAOM: National Mycological Herbarium.

## Authors' contributions

JM performed confocal imaging and aphidicolin experiments, analyzed data and contributed in writing the manuscript. YD performed spore development experiments and contributed in writing the manuscript. MSA designed experiments on AMF extraradical phases and analyzed the data. MH (corresponding author) designed experiments, evaluated the data, coordinated the research project, constructed the figures and wrote the manuscript. All of the authors read and approved the final manuscript.

## Supplementary Material

Additional file 1 **Example of a z-stack series of *Glomus diaphanum *live spore observed with a confocal microscope**. Nuclei were visualized as large green spots with SytoGreen fluorescent dye, while mitochondria are shown as small green spots. The movie was acquired at 1 frame every 0.15 μm for a total of 260 frames and displayed at a rate of 5 frames/sec. Click here for file

Additional file 2 **3D reconstruction of 260 optical sections with z-resolution of 0.15 μm, showing a full turn of *G. diaphanum *live spore with nuclei color indexed**. The movie is displayed at a rate of 5 frames/sec. Click here for file

Additional file 3 **Merged image of 100 optical sections of sister spores of *G. diaphanum *showing heterogeneity of nuclear content**. Nuclei were stained with SytoGreen fluorescent dye. Scale bar represents 47.62 μm. Click here for file

Additional file 4 **The mean number of newly produced spores plotted against time (days) of *G. irregulare *treated with aphidicolin diluted in DMSO or DMSO alone as a control on six replicates each**. Red and blue curves represent aphidicolin and control treatments, respectively. Click here for file

Additional file 5 **The number of nuclei per spore plotted against the diameter of spores of *G. irregulare *treated with aphidicolin (red squares, n = 22) and the control (blue triangles, n = 68)**. Click here for file

Additional file 6 **Time-lapse series on *G. diaphanum *live spore**. Nuclei were visualized as large green spots with SytoGreen fluorescent dye, while mitochondria were stained with MitoTracker and are shown as small red spots. The movie was acquired at 1 frame every 5 min for a total of 90 min and displayed at a rate of 5 frames/sec. Click here for file

Additional file 7 **Merged image of 150 optical sections of non-germinating spore of *G. diaphanum *showing nuclei in a degenerating phase**. Nuclei were stained with SytoGreen fluorescent dye. Scale bar represents 19.71 μm. Click here for file

Additional file 8 ***G. diaphanum *spore observed with confocal microscope where nuclei were visualized by SytoGreen fluorescent dye (green spots) showing direct connection of spore and the subtending hyphal cytoplasm (arrows)**. A, xy focal plane; B, yz projection; and C, xz projection. Scale bar in panel A represents 17.93 μm. Click here for file

Additional file 9 **AMF spore development of *G. irregulare *over time, viability and germination rates**. Click here for file

Additional file 10 **Cell wall structure of *G. intraradices *spores of different ages**. A, Open 30 day-old spore with subtending hypha, outside hyaline spore wall and inside pale yellow pigmented spore wall. B, Closer view of 60 day-old spore wall with bi-layered outer walls and laminated inner wall. C, Walls of 90 day-old mature spore with bi-layered outer wall and multi-laminated inner wall. Images were taken with DIC optical microscope. Scale bars represent 20 μm. Click here for file
