# Correction: Spore development and nuclear inheritance in arbuscular mycorrhizal fungi

**DOI:** 10.1186/1471-2148-11-97

**Published:** 2011-04-14

**Authors:** Julie Marleau, Yolande Dalpé, Marc St-Arnaud, Mohamed Hijri

**Affiliations:** 1 Université de Montréal, Département de sciences biologiques, Institut de recherche en biologie végétale, 4101 rue Sherbrooke Est, QC, H1X 2B2, Canada; 2 Agriculture and Agri-Food Canada, 960 Carling Ave. Ottawa, On, K1A 0C6, Canada

## Correction

After the publication of this work [1], we found that the equations of linear regressions in the section "Live cell imaging of AMF spores" didn't correspond to those shown in figure two (Figure 1 in this article) which was due to an inversion of the axis during the revision. Figure two shows regressions of the spore diameter (y) plotted against the number of nuclei in juvenile and mature spores (x), while in the text we showed equations of linear regressions of the number of nuclei (y) plotted against the spore diameter (x) for each AMF species. The inversion of the axis didn't affect our conclusions.

**Figure 1:**
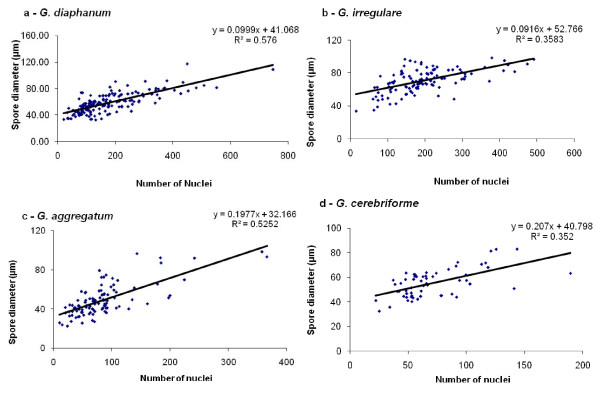
**Figure two: Number of nuclei per spore**. The spore diameter of juvenile and mature spores (y) plotted against the number of nuclei in spore (x). *G. cerebriforme *spores had the smallest number of nuclei per spore and were also the smallest in size, while *G. diaphanum *spores had the largest number of nuclei per spore. There is a positive linear relation between the number of nuclei per spore and spore diameter: **A**, *G. diaphanum *(y = 0.0999x + 41.068, R^2 ^= 0.576); **B**, *G. irregulare *(y = 0.0916x + 52.766, R^2 ^= 0.358); **C**, *G. aggregatum *(y = 0.1977x + 32.166, R^2 ^= 0.525); and **D**, *G. cerebriforme *(y = 0.207x + 40.798, R^2 ^= 0.352). All slopes were statistically significant at *p *< 0.05.

We would like to correct equations of these linear regressions in the text in page 3 as follow:

We found a positive linear relation between the number of nuclei in a given spore and its diameter for all AMF taxa: *Glomus diaphanum *(y = 0.0999x + 41.068, R^2 ^= 0.576), *Glomus irregulare *(y = 0.0916x + 52.766, R^2 ^= 0.358), *Glomus aggregatum *(y = 0.1977x + 32.166, R^2 ^= 0.525) and *Glomus cerebriforme *(y = 0.207x + 40.798, R^2 ^= 0.352). All slopes were statistically significant at *p *= 0.05.

We apologise for any inconvenience that this inaccuracy in presentation of the data used in the article [1] might have caused.
