# Supplementary figures and images for: Spore development and nuclear inheritance in arbuscular mycorrhizal fungi
Source: BMC Evol Biol. 2011 Feb 24;11:51. doi: 10.1186/1471-2148-11-51 (PMC3060866; doi:10.1186/1471-2148-11-51)

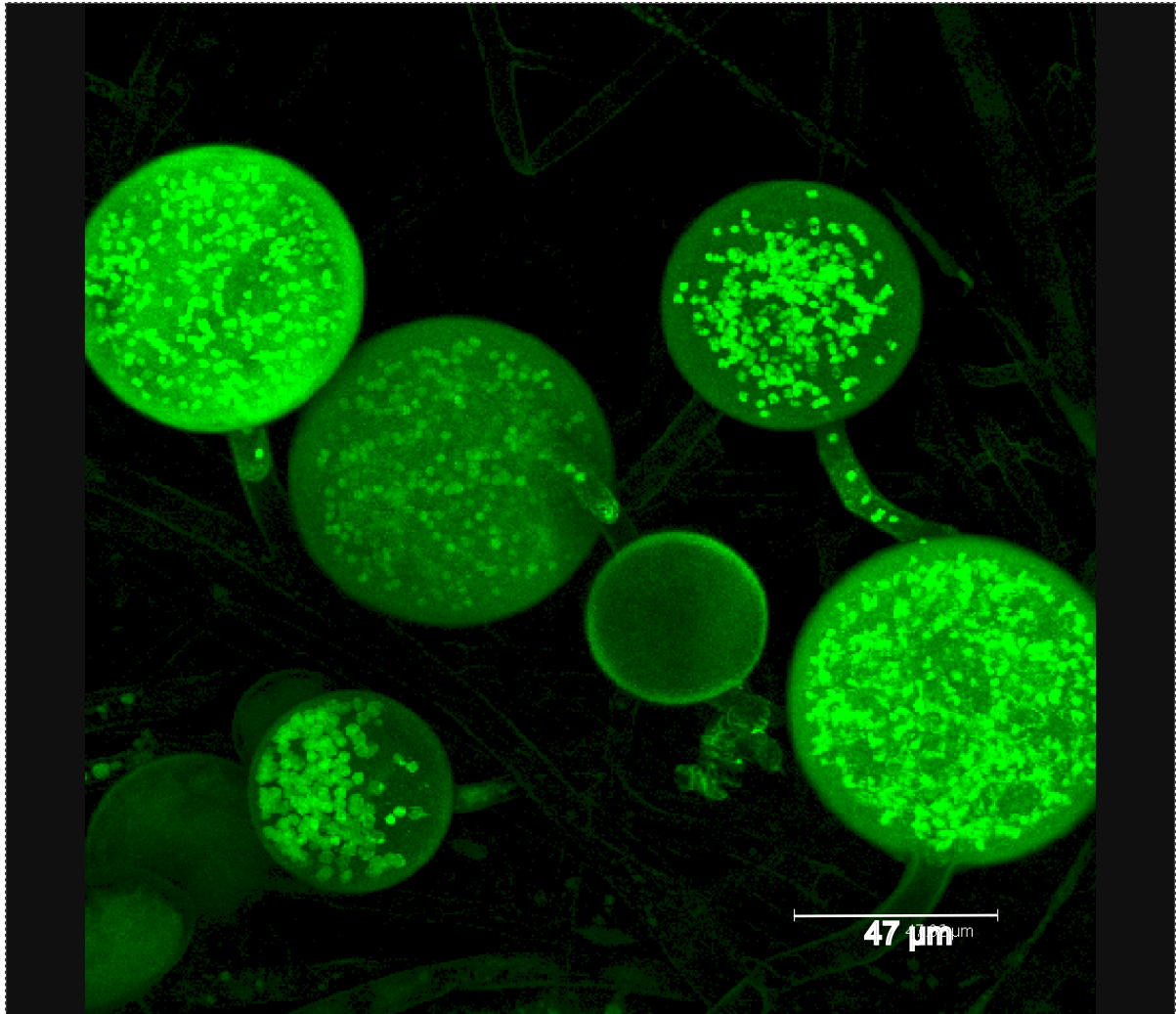

Supplement: Additional file 3 — Merged image of 100 optical sections of sister spores of G. diaphanum showing heterogeneity of nuclear content. Nuclei were stained with SytoGreen fluorescent dye. Scale bar represents 47.62 μm. [file 1471-2148-11-51-S3.PDF]

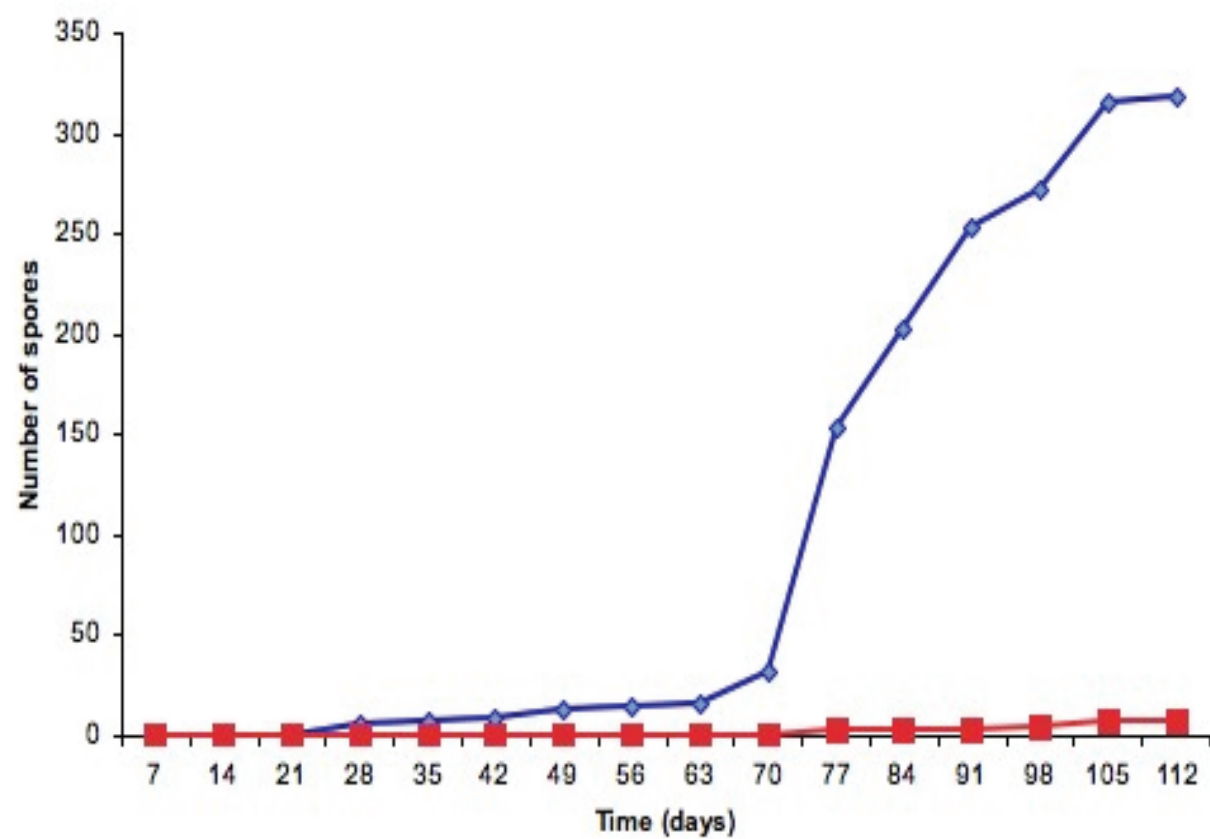

Supplement: Additional file 4 — The mean number of newly produced spores plotted against time (days) of G. irregulare treated with aphidicolin diluted in DMSO or DMSO alone as a control on six replicates each. Red and blue curves represent aphidicolin and control treatments, respectively. [file 1471-2148-11-51-S4.PDF]

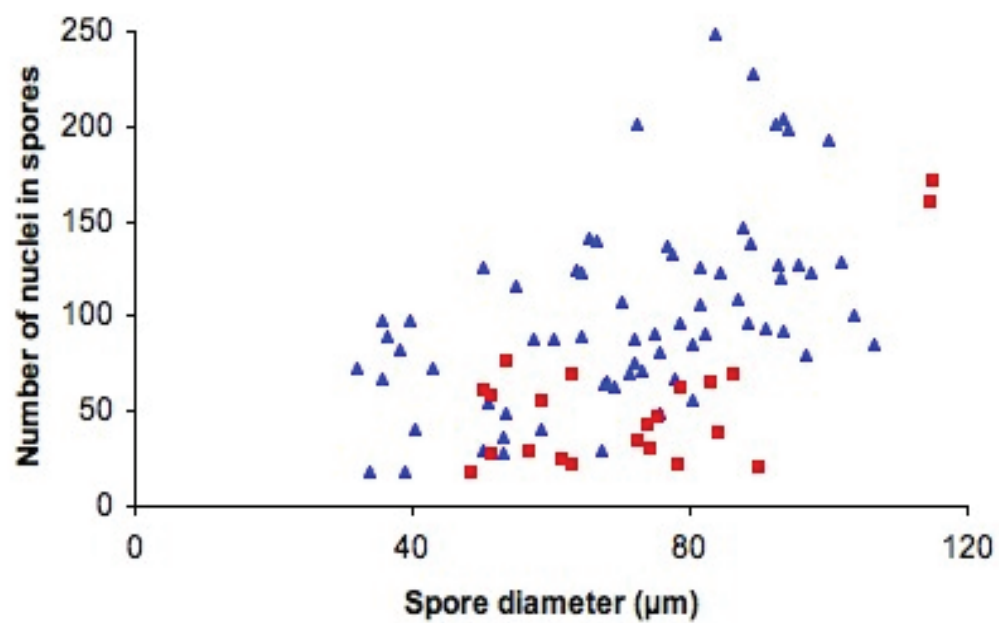

Supplement: Additional file 5 — The number of nuclei per spore plotted against the diameter of spores of G. irregulare treated with aphidicolin (red squares, n = 22) and the control (blue triangles, n = 68). [file 1471-2148-11-51-S5.PDF]

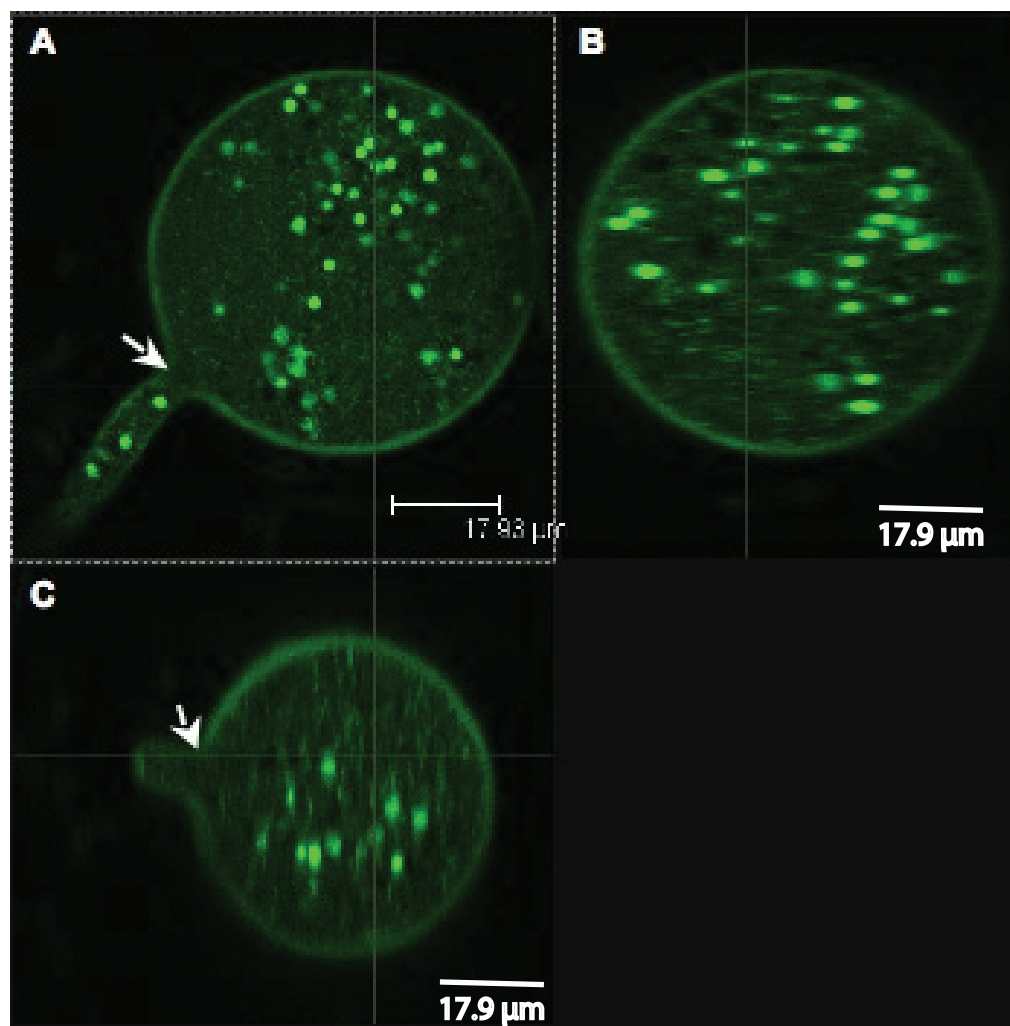

Supplement: Additional file 7 — Merged image of 150 optical sections of non-germinating spore of G. diaphanum showing nuclei in a degenerating phase. Nuclei were stained with SytoGreen fluorescent dye. Scale bar represents 19.71 μm. [file 1471-2148-11-51-S7.PDF]

Additional file 8

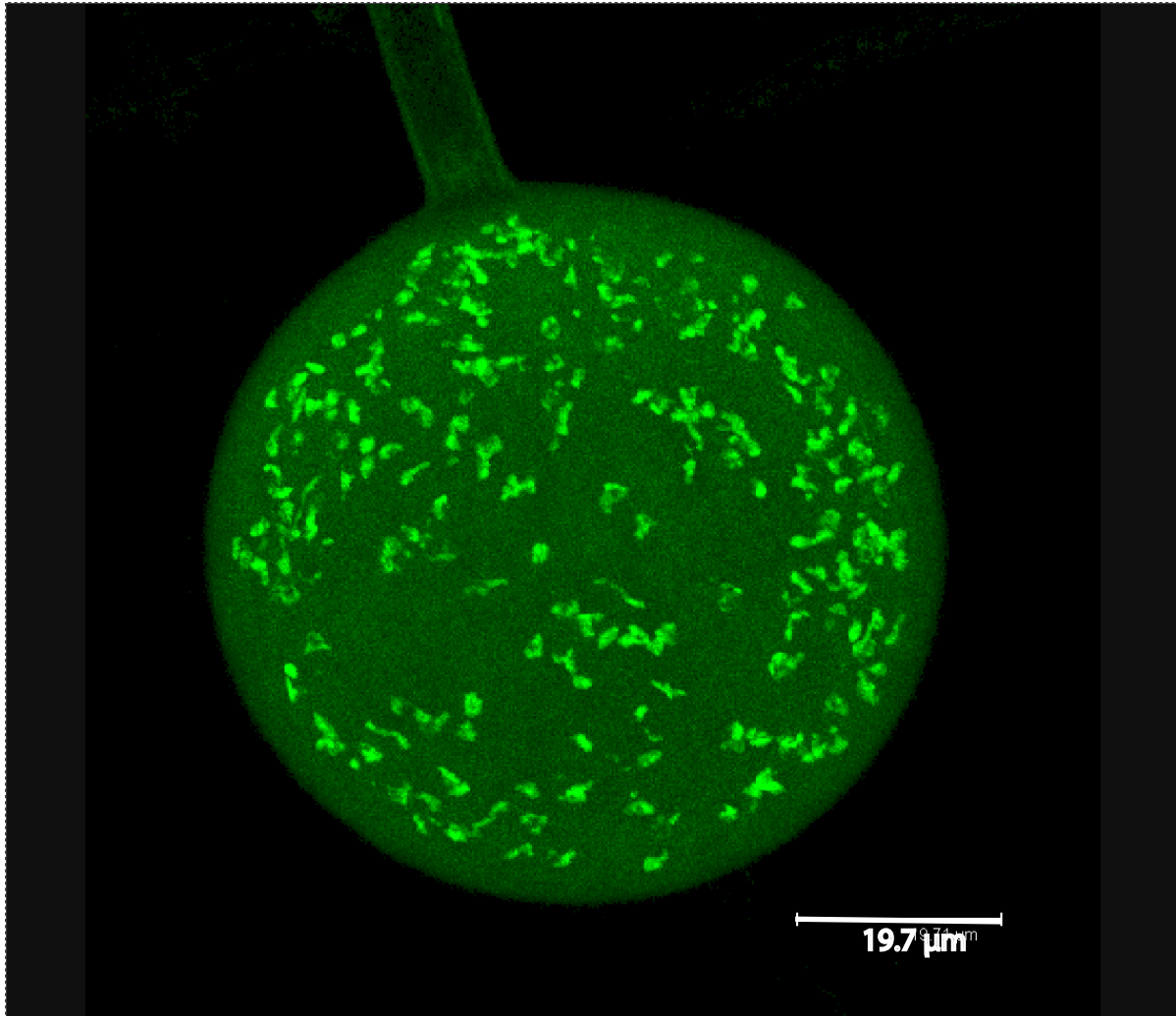

Supplement: Additional file 8 — G. diaphanum spore observed with confocal microscope where nuclei were visualized by SytoGreen fluorescent dye (green spots) showing direct connection of spore and the subtending hyphal cytoplasm (arrows). A, xy focal plane; B, yz projection; and C, xz projection. Scale bar in panel A represents 17.93 μm. [file 1471-2148-11-51-S8.PDF]

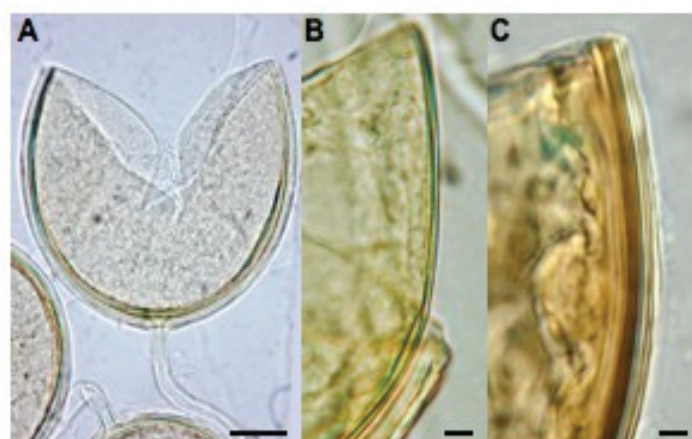

Supplement: Additional file 10 — Cell wall structure of G. intraradices spores of different ages. A, Open 30 day-old spore with subtending hypha, outside hyaline spore wall and inside pale yellow pigmented spore wall. B, Closer view of 60 day-old spore wall with bi-layered outer walls and laminated inner wall. C, Walls of 90 day-old mature spore with bi-layered outer wall and multi-laminated inner wall. Images were taken with DIC optical microscope. Scale bars represent 20 μm. [file 1471-2148-11-51-S10.PDF]
