# Supplementary material for: Spore development and nuclear inheritance in arbuscular mycorrhizal fungi
Source: BMC Evol Biol. 2011 Feb 24;11:51. doi: 10.1186/1471-2148-11-51 (PMC3060866; doi:10.1186/1471-2148-11-51)
Supplement: Additional file 9 — AMF spore development of G. irregulare over time, viability and germination rates. [file 1471-2148-11-51-S9.DOC]

**Additional file 9 - Supplementary text and references**

**AM fungal spore development of *G. irregulare* over time, viability and germination rates.**

AM fungi were propagated under *in vitro* conditions on root-organ cultures. Gel plugs (10 mm diameter) from a growing culture of *G. irregulare* (DAOM 197198) bearing spores and mycelium were transferred to Petri plates containing a Minimal (M) medium (1 solidified with 4g L-1 Gel Gro TM (ICN Biomedicals, Inc, Irvine, California USA), at the rate of one plug per plate for a total of 10 plates. In each bi-compartmented Petri plate, a *Daucus carota* L. transformed root was deposited in the vicinity of the gel plug and inverted cultures were incubated at 28°C in darkness until propagation in the distal section of the culture.

*Spore selection and measurement over time.* The diameter of globose juvenile to mature *G. irregulare* spores isolated from field soil ranged from 50 to 130 μm with a mean value of 92-95 μm [1]. *In vitro* differentiated spores reached a similar range of spore diameter. The original description of *G. irregulare* Blaszk., Wubet, Renker & Buscot (2009) [1] characterized spores as ovoid, to irregular, pale yellow, 60-130 X 80-240 µm in diameter, surrounded by two hyaline semi-permanent walls covering a laminated inner wall reactive to Melzer's reagent.

Architectural hyphae of *G. irregulare* colonies, also called runner hyphae and their ramifications of 7-12 μm diameter are surrounded by a bi-layered wall (1.0 - 1.8 μm thick). Intercalary swellings along those hyphae give rise to spore primordia (Fig. 5A). Their early detection was considered to be time 0 of spore ontogeny observations. The spore primordia rapidly increase in diameter, fed by a continuous cytoplasm flow (Fig. 5C). This corresponds to stage 1 of spore differentiation [2]. At 15 days old, still attached to their mother hyphae, juvenile spores, 24-36 μm in diameter, usually differentiated a thin inner wall in addition to their original double hyphal wall, for a total wall thickness of 1.5 μm (Fig. 5C). The new differentiated inner wall corresponds to the first lamination of the laminated wall seen on mature spores. This corresponds to the beginning of stage 2 of spore differentiation [3]. Concurrently to inner wall differentiation, spores continue to increase in diameter via the constant inflow from the fungal colony through the mother hyphae. Between 15 and 30 days, 3 to 4 laminations are newly cumulated to the inner spore wall. Spore diameter doubled to attain 59-70 μm, with a spore wall between 2.3 and 3.1 μm thick. At the proximal side of the spore, the subtending hyphae that link the spore to the fungal colony slowly compacted from flare to cylindrical shape and were gradually lined at the pore level by the newly differentiated inner wall laminations (Fig. S6A). After 30 days of growth, subsequent spore development consists essentially of the synthesis of additional laminations (Additional File 10A) and the slight thickening of the median wall. At 60 days old, spores attained almost their maximum size (77-115 μm in diameter). On crushed spores, outer and median walls readily separated from the inner laminated wall (Additional File 10B) and often appeared as an empty hyaline shell. With spore maturation, up to twelve successive laminations can be detected in 90 day-old spores (Additional File 10C). With age, spore volume increase slowed down with as a result the gradual compaction of the newly differentiated laminations of the inner spore wall.

**References**

1. Blaszkowski J, Czerniawska B, Wubet T, Schäfer T, Buscot F: ***Glomus irregulare*, a new mycorrhizal fungus in the Glomeromycota**. 2009. *Mycotaxon* 106: 247-267.

### 2. Friese CF, Allen MF: The spread of VA mycorrhizal fungal hyphae in the soil: inoculum types and external hyphal architecture. *Mycologia* 1991, 83(4):409-418.

### 3. Sturmer SL, Morton JB: Developmental patterns defining morphological characters in spores of four species in *Glomus*. *Mycologia* 1997, 89(1):72-81.
